# Automated Analysis of Digitized Letter Fluency Data

**DOI:** 10.3389/fpsyg.2021.654214

**Published:** 2021-07-29

**Authors:** Sunghye Cho, Naomi Nevler, Natalia Parjane, Christopher Cieri, Mark Liberman, Murray Grossman, Katheryn A. Q. Cousins

**Affiliations:** ^1^Linguistic Data Consortium, University of Pennsylvania, Philadelphia, PA, United States; ^2^Penn Frontotemporal Degeneration Center, University of Pennsylvania, Philadelphia, PA, United States

**Keywords:** neuropsychological test, automated speech analysis, verbal retrieval, executive function, verbal fluency, phonetic similarity

## Abstract

The letter-guided naming fluency task is a measure of an individual’s executive function and working memory. This study employed a novel, automated, quantifiable, and reproducible method to investigate how language characteristics of words produced during a fluency task are related to fluency performance, inter-word response time (RT), and over task duration using digitized F-letter-guided fluency recordings produced by 76 young healthy participants. Our automated algorithm counted the number of correct responses from the transcripts of the F-letter fluency data, and individual words were rated for concreteness, ambiguity, frequency, familiarity, and age of acquisition (AoA). Using a forced aligner, the transcripts were automatically aligned with the corresponding audio recordings. We measured inter-word RT, word duration, and word start time from the forced alignments. Articulation rate was also computed. Phonetic and semantic distances between two consecutive F-letter words were measured. We found that total F-letter score was significantly correlated with the mean values of word frequency, familiarity, AoA, word duration, phonetic similarity, and articulation rate; total score was also correlated with an individual’s standard deviation of AoA, familiarity, and phonetic similarity. RT was negatively correlated with frequency and ambiguity of F-letter words and was positively correlated with AoA, number of phonemes, and phonetic and semantic distances. Lastly, the frequency, ambiguity, AoA, number of phonemes, and semantic distance of words produced significantly changed over time during the task. The method employed in this paper demonstrates the successful implementation of our automated language processing pipelines in a standardized neuropsychological task. This novel approach captures subtle and rich language characteristics during test performance that enhance informativeness and cannot be extracted manually without massive effort. This work will serve as the reference for letter-guided category fluency production similarly acquired in neurodegenerative patients.

## Introduction

Letter-guided verbal fluency tasks (sometimes referred to as phonemic fluency) are frequently administered to measure an individual’s executive function, working memory, and lexical access. Executive function drives a search through the lexicon (lexical access) in order to find target words, and working memory helps keep track of words already mentioned so that responses are not repeated. Typically, participants are asked to list words that start with a certain letter (e.g., “f,” “s,” and “a”) in a letter-guided fluency task during a limited time period (e.g., 30 s and 1 min); the final fluency score is the tally of words produced during the task. In addition to letter-guided, category-guided fluency (sometimes referred to as semantic fluency) is an alternative fluency task where participants are asked to produce words that belong to a certain semantic category (e.g., animals or tools). In clinical settings, the fluency score has been shown to be sensitive to diverse types of neurodegenerative disease, psychosis, and many other neurological conditions (Elvevåg et al., 2002; Forbes-Mckay et al., 2005; Rascovsky et al., 2007; Libon et al., 2009; Juhasz et al., 2012; Cook et al., 2014; Van Den Berg et al., 2017; Lebkuecher et al., 2021).

While total fluency score is often used to estimate executive functioning and lexical access ability (Amunts et al., 2020; Beatty-Martínez et al., 2020), performance may be affected by various factors. First, lexical characteristics of words produced during the task may be correlated with performance; higher scores have been associated with word production of lower frequency and higher AoA (e.g., Forbes-Mckay et al., 2005; Venneri et al., 2008). Second, phonetic and semantic relations among words produced are highly correlated with fluency performance, with more clustering (producing words within the same phonetic or semantic subcategory) and switching (the frequency of shifting clusters) predicting better performance (Troyer et al., 1997). Third, timing within task has also received attention in the literature. Previous studies found that the number of words produced during the initial 30 s is greater than the number of words during the last 30 s in a 1-min fluency test (Crowe, 1998; Fernaeus and Almkvist, 1998; Sauzéon et al., 2011). Luo et al. (2010) also showed that the number of words exponentially decreases over time during the task when words are binned by 5 s. Similarly, Özdemir and Tunçer (2021) showed that the mean number of words decreased by timing during the task in semantic fluency tasks among elderly participants. Finally, response time (RT) is a sensitive measure of both verbal and executive control functioning in fluency tasks, and it is significantly correlated with fluency performance (e.g., Rohrer et al., 1995; Luo et al., 2010; Shao et al., 2014). For example, Shao et al. (2014) showed that vocabulary access ability was negatively correlated with the first-response RT (i.e., the pause duration from the beginning of the task to the onset of the first word). Luo et al. (2010) observed that the mean inter-word RT (i.e., the average pause duration from the onset of the first word to the onset of the subsequent words) is more sensitive to executive control ability than the first RT. Importantly, these fluency metadata can also be applied to patient populations. For example, Forbes-Mckay et al. (2005) showed that higher frequency, shorter length, and lower age of acquisition (AoA) during category fluency tasks distinguish patients with Alzheimer’s disease from healthy controls.

Despite these rich and complex characteristics of fluency data, many clinical settings continue to use only the final tally of produced words to capture a fluency score. A comprehensive and systematic analysis of letter fluency metadata is still lacking, due in part to reliance on manual methods in many previous fluency task analyses that are lengthy, burdensome, require some level of expertise, and therefore cannot be easily applied on a large scale. For example, semantic and phonetic relations among the words produced during the tests were qualitatively defined, and thus judgments such as these are subjective and may have difficulty with reproducibility and reliability. Likewise, there are limitations to qualitative assessments of transcribed data. For example, phonetic relations among the words transcribed do not necessarily reflect real-world pronunciation. Phonological rhyming, which is often used to qualitatively define phonetic subcategories, may differ from speaker to speaker depending on the dialect, the socioeconomic level, and demographic characteristics of speakers, such as age and sex (Bowey, 1995; Cox and Palethorpe, 2011; Welcome and Alton, 2015; Gerwin et al., 2019). Similarly, qualitative grouping of semantic categories can be subjective and inconsistent depending on how raters define semantic subcategories. Importantly, to accurately indicate the degree of similarity between words, phonetic or semantic difference between words must be measured with a gradual, continuous scale, rather than a discrete scale.

Recent developments in language technology make possible the large-scale analyses of fluency metadata and can address the limitations of previous manual methods. Because they are quantitative, objective, consistent, and automated, they can be accomplished quickly, reliably, and with minimal effort. Also, because they are automated, they offer the promise of removing inconsistencies across studies as a confounding factor in comparing analyses of performance. Our group has previously used these fully automated and easily reproducible methods to characterize speech during a semi-structured speech sample obtained during a picture description (e.g., Nevler et al., 2017, 2019; Cho et al., 2021a,b).

In this paper, we applied these automated analyses for the first time to characterize acoustic, lexical, phonetic, and semantic features of words produced during a common neuropsychological measure, the letter fluency task, in healthy, and young speakers. We focus on F-letter fluency since this is one of the most frequently administrated tasks in many clinical settings. In this study, we investigated three aspects of F-letter fluency (total score, RT, and timing within task) and test how these correlate with language characteristics: word frequency, AoA, word familiarity, semantic ambiguity, concreteness, number of phonemes in a word, number of syllables in a word, articulation rate, word duration, and phonetic and semantic distances between two words. The following are our hypotheses:

1. Total Score: We hypothesize that fluency performance will correlate with AoA and word frequency, as observed previously (Forbes-Mckay et al., 2005), and that individuals who produce less familiar and longer words will have higher total fluency scores. We also hypothesize that faster articulation rates and higher phonetic similarity between words will relate to better performance. In addition, we hypothesize that total score will be associated with higher variance within language features, that is, individuals who are able to produce both low- and high-frequency words will perform better than individuals who preferentially produce high-frequency words.
2. RT: We hypothesize that language characteristics of words produced affect first and inter-word RT in that RT will increase before less frequent, longer, and later-acquired words. We also hypothesize that inter-word RT between two adjacent F-letter words will increase if the two words are less phonetically or semantically similar.
3. Timing within task: We hypothesize that language characteristics of words produced will change over time during the task and that at the beginning of the task speakers will produce words that can be easily accessed from the lexicon compared to later in the task. Thus, we expect that AoA and number of phonemes will increase over time, whereas word frequency will decrease.

## Materials and Methods

### Participants

We recruited 82 undergraduate students in total for the study, but six participants were excluded from the analysis due to missing demographic information. Out of the remaining 76 participants, 35 were female and the others were male speakers. The participants were matched on age and education level. Table 1 shows demographic characteristics of the 76 participants.

**Table 1:** Mean (standard deviation) of demographic characteristics of the participants by sex.

	*F* (*N* = 35)	*M* (*N* = 41)	*p*-value
Age (years)	19.9 (1.0)	20.2 (0.9)	0.147
Education (years)	13.4 (1.0)	13.7 (0.9)	0.147
Fluency score	11.2 (2.6)	11.6 (3.2)	0.549

*F, female; M, male*.

### Data Collection

The participants volunteered to participate in a 30-min study, where they performed three neuropsychological tests, including the F-letter fluency task, and four different picture description tasks in a randomized order. All tasks were digitally recorded with a sampling rate of 16,000 Hz in a soundproof recording booth. The experiment was web-based and self-paced. Prior to the experiment, written instructions were presented to participants on their screen. Participants then initiated each task by pressing a button, at which point participants were shown the letter (e.g., “f”) for their guided fluency task or a picture for the picture description task and recording began. In this report, we examined only the F-letter fluency task, where the participants were asked to name as many unique words starting with “f” as possible within 30 s, while omitting proper nouns and numbers. All participants read the same instructions. A shortened version of this self-paced, web-based experiment is available at https://speechbiomarkers.org. The prerequisites for participating in this study were to not have any hearing or speaking difficulties and to be a native speaker of English. The participants were not asked if they had any writing or reading difficulties, but all participants were able to read the instructions on the screen and complete the tasks successfully. The participants received course credit for their participation, and this study was approved by the Institutional Review Board at the University of Pennsylvania.

Trained annotators at the Linguistic Data Consortium (LDC) of the University of Pennsylvania generated verbatim transcripts of all recordings following standardized transcription guidelines (first-pass), and the generated transcripts were further reviewed and corrected by senior transcribers who had years of experience of the LDC transcription process for quality check (second-pass). The purpose of the current second-pass transcription pipeline was to generate the most accurate and consistent transcripts possible; therefore, agreement rate between annotators was not calculated. The annotators transcribed all types of non-verbal vocalizations, such as breath, laugh, cough, lip smack, and interjections (*um, uh*, and *er*) with standard spellings following project guideline. They were also instructed to transcribe partial words with an attached single hyphen (e.g., *fi*-) to generate consistent transcripts.

### Data Processing and Measurements

In order to automatically calculate F-letter fluency scores, we first tagged part-of-speech (POS) categories of all words with the POS tagger in spaCy (Honnibal and Johnson, 2015), a natural language processing library in Python. To maximize accuracy, the automatically generated POS categories were manually inspected and corrected. The automated POS tagging and forced alignment had good performance with minimal corrections required. We corrected the POS category of 12 words out of 986 words in total (1.2%). We used this POS tagging information to subtract the number of proper nouns (e.g., *Fanta*; 4 out of 986 words), numbers (e.g., *five* and *fifteen*; 16 out of 986 words), and repeated lemmas (28 out of 986 words (2.8%); ranges: 0–5 words; mean = 0.34 ± 0.78 words per participant) tagged by spaCy and to automatically calculate the number of F-letter words for each participant. We checked repeated lemmas to make sure the repeated words were not proper nouns or numbers that were already excluded to avoid the subtraction of the same word twice. The average F-letter score during 30 s for the participants is shown in Table 1.

We aligned audio signals with the verbatim transcripts using a forced aligner that was developed at LDC, which generated frame-wise timestamps of words and silence between words. As with POS, all alignments were visually inspected to ensure accuracy. Either the start or end boundary of 71 out of 986 words was manually corrected (7.2%) by a trained linguist. We measured the start time, duration, and RT of all words using the timing information in the alignment files. We followed previous studies for the distinction of the first RT from the rest RTs. In this study, the first RT refers to the pause duration from the beginning of the task to the onset of the first F-letter word, and inter-word RTs refer to the pause duration from the offset of a previous F-letter word to the onset of the next F-letter word. Since we were interested in cognitive processing time and its relation with language characteristics of individual F-letter words, RT included the duration of filled pauses (e.g., *um* and *uh*), partial words (e.g., *f*-, *fi*-, and *fu*-), and non-verbal vocalizations, such as lip smack and laugh. The number of participants who produced 0 to 3 fillers and partial words during the task was the majority (47 out of 76; mean = 3.7 ± 3.4 words), and the number of intermediate words was not significantly correlated with F-letter scores (*p* = 0.54). We note that the definition of inter-word RTs in this study is slightly different from the definition of subsequent latency in previous studies (Rohrer et al., 1995; Luo et al., 2010; Shao et al., 2014), which was the duration of the onset of the first word to the onsets of subsequent words.

We rated F-letter words for concreteness (1: most abstract and 5: most concrete; Brysbaert et al., 2014), semantic ambiguity (number of different meanings of a word in a given context; Hoffman et al., 2013), word frequency (log_10_-scaled frequency per million words; Brysbaert and New, 2009), AoA (the average age at which people acquire a given word; Brysbaert et al., 2018), and word familiarity [percent of people who answer they know a given word (in z-score); Brysbaert et al., 2018], using published norms. We determined the number of phonemes and syllables of all words using the CMU pronouncing dictionary (Carnegie Mellon Speech Group, 2014) within the Natural Language Toolkit (NLTK; Loper and Bird, 2002) package in Python. We measured articulation rate for each speaker [= total number of syllables/total duration of all words; syllable per second (sps)], using the timestamps from the forced aligner and the number of syllables from NLTK. Our method was previously published (Cho et al., 2021a,b).

We measured phonetic and semantic distances between two consecutive F-letter words to examine how phonetic and/or semantic similarity between words affects total score and inter-word RT and how phonetic and semantic distances change over time during the task. Phonetic distance (i.e., how similar or dissimilar the pronunciations of two adjacent words are) between F-letter words was computed with the dynamic time warping (DTW) algorithm (e.g., Sakoe and Chiba, 1978; Berndt and Clifford, 1994) using the DTW-python library (Giorgino, 2009) in Python. The DTW algorithm aligns two time-series signals in different length by either stretching or compressing one signal to match the other and outputs the remaining cumulative distance between the two signals. We used the cumulative distance as a metric of phonetic distance. First, we extracted the first to the 13th mel-frequency cepstral coefficients (MFCCs) from two consecutive F-letter words. The mel-frequency cepstral coefficients capture acoustic properties of the phonemic representation of a word, and they are the most frequently used acoustic features in modern automatic speech recognition systems. After aligning the 13 obtained MFCCs of the two consecutive F-letter words using DTW, we calculated the normalized Euclidean distance between those matrices. The obtained Euclidean distance is a metric of phonetic similarity between two F-letter words (the smaller the distance and the more similar the word pairs).

Semantic distance was calculated as the Euclidean distance between word vectors of two F-letter words in sequence using a pre-trained word vector representation of GloVe^1^ (Pennington et al., 2014), where the meaning of a word was represented as a 300 dimensional vector. GloVe’s word representations were computed by calculating how frequently words occurred together with one another in large-scale corpora and by reducing the dimension of word co-occurrence information into 300 dimensions using a dimensionality reduction algorithm. We used GloVe’s largest pre-trained model, which was trained with 2.2 million words, in order to include as many F-letter words as possible in our data. In total, we had 836 F-letter word pairs, and seven word pairs were excluded from the analysis of semantic distance, since the largest GloVe model did not include the word representation of one word in those pairs.

### Statistical Considerations

First, we examined the relations among F-letter scores, the first and inter-word RTs, and timing within task in order to validate that our data matched previous findings. We correlated F-letter scores to the first RTs and the mean inter-word RTs with a Spearman’s correlation test, since the data did not meet assumptions for parametric tests. The relation between inter-word RTs and timing within task was tested with a linear mixed-effects model, where individual speakers were treated as a random effect [inter-word RT ~ timing within task + (1 |speaker)]. We did not test the relation between the first RT and timing within task, since the estimated coefficient for this relation is always one. To measure the relation of timing within task and the number of F-letter words, we sliced the task into three 10-s time windows to count the number of F-letter words per 10-s interval (beginning 0–10 s, middle 10–20 s, and end 20–30 s) and performed ANOVA and Tukey’s post-hoc test, since the data met assumptions for parametric tests.

Spearman’s correlations tested the association between each language measure (averaged over all words for a participant) and F-letter fluency performance. We also performed Spearman’s correlation tests to examine relations of F-letter scores to averaged word duration and articulation rate (syllables per second).

Since multiple variables showed significant correlations with fluency performance, a linear regression model using dimension reduction identified which variables most contributed to F-letter performance: F-letter score was included as dependent variable and all language measures that showed significant correlations with F-letter scores were included as independent variables (score ~ mean values of language variables). A stepwise backward elimination approach was implemented in the linear model in order to find the best fit model. We also reported a correlation matrix of all explanatory variables.

In addition to testing an individual’s average performance, we investigated how variability within an individual for each of our language measures was related to the F-letter fluency performance. Individualized standard deviation (*SD*) for each measure was calculated, and Spearman’s correlation tested associations with fluency score. As above, a linear regression model examined how variability within significant language measures affected the F-letter performance (score ~ *SD* values of the language variables), and a stepwise backward elimination approach was implemented to find the best fit model. A correlation matrix of the *SD* values of all variables was also reported. We did not build a model with both the average and *SD* values of all variables, since the mean and *SD* values of some variables were highly correlated.

In order to investigate the relations of our language measures to inter-word RTs and timing within task, separate linear mixed-effects models regressed the language measures to inter-word RTs and timing within task, respectively, where our language measures were the independent variables, RT, or timing within task were the dependent variables, and the random slope for each language variable and individual speaker was included [RT or task timing ~ the target language variable + (language variable | speaker)]. Separate linear regression models tested the relation of the first RT and the language characteristics of the first F-letter word, except phonetic and semantic distances, since those metrics measured the phonetic/semantic distance between two F-letter words. All statistical analyses were performed in RStudio Team (2020) version 1.3.959, and the linear mixed-effects models were built with the lme4 (Bates et al., 2015) and lmerTest packages (Kuznetsova et al., 2017).

## Results

### F-Letter Scores, RT, and Task Time

**Figure 1:**
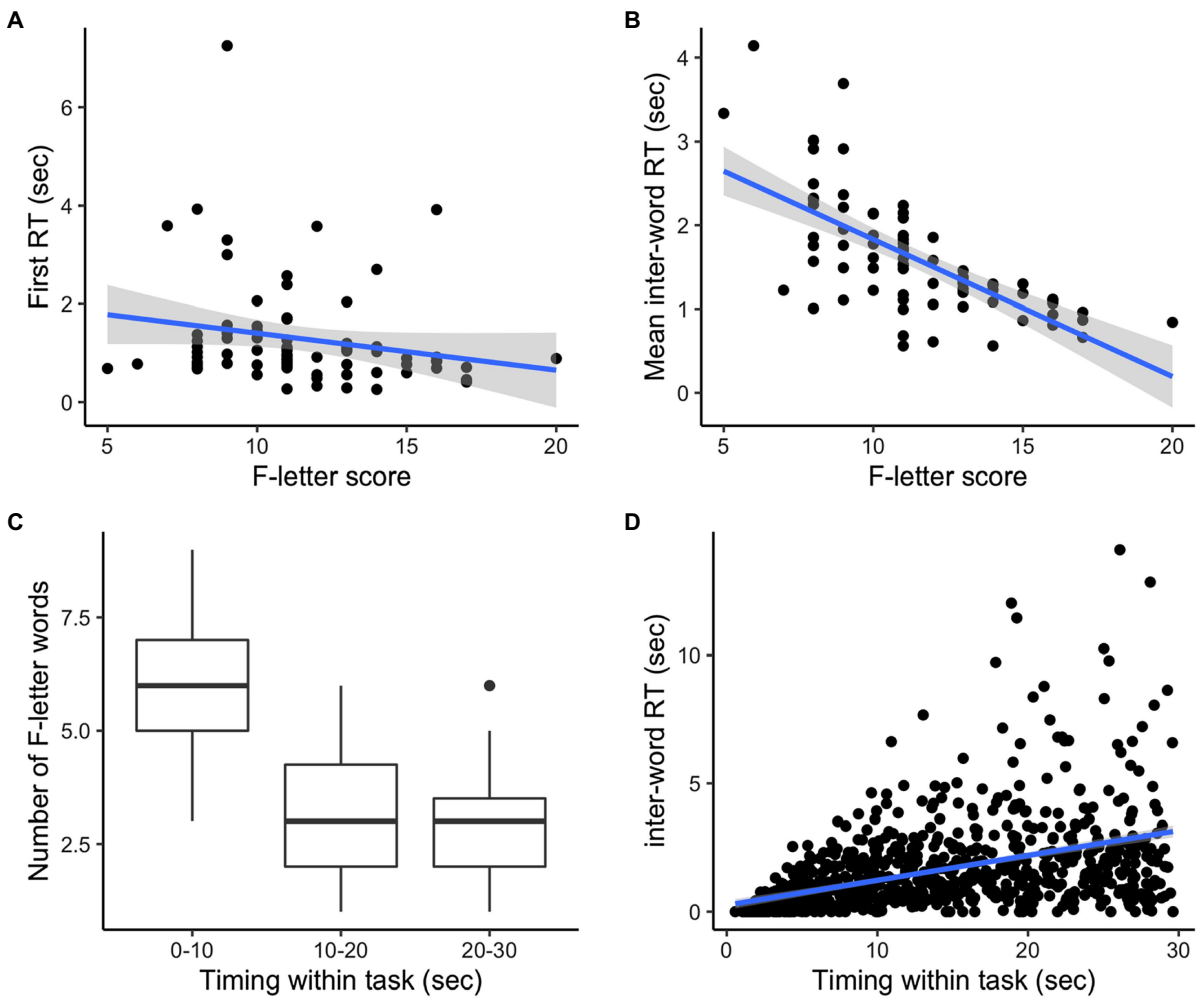
Relations of F-letter performance, RT, and timing within task.

First RT was negatively correlated with fluency score (*ρ* = −0.27, *p* = 0.017; Figure 1A); the correlation remained significant when one outlier participant (first RT > 6 s) was excluded (*ρ* = −0.26, *p* = 0.025). There was a strong negative correlation between the mean inter-word RT and fluency score (*ρ* = −0.73, *p* < 0.001; Figure 1B). Thus as expected, speakers who took longer to produce their first and subsequent words scored lower in the task. When examining time within the task, speakers tended to produce more F-letter words during the first 10 s than the second or the last 10 s [*F* (2,220) = 94.54, *p* < 0.001 for both comparisons; Figure 1C]. The number of F-letter words during the second 10 s was not significantly different from the last 10 s (*p* = 0.077). Lastly, RT slowed by an average of 0.09 s per 1 s of the task timing [β = 0.09, *t* (781.5) = 16.27, *p* < 0.001; Figure 1D].

### F-Letter Scores and the Language Measures

#### Similarity

**Figure 2:**
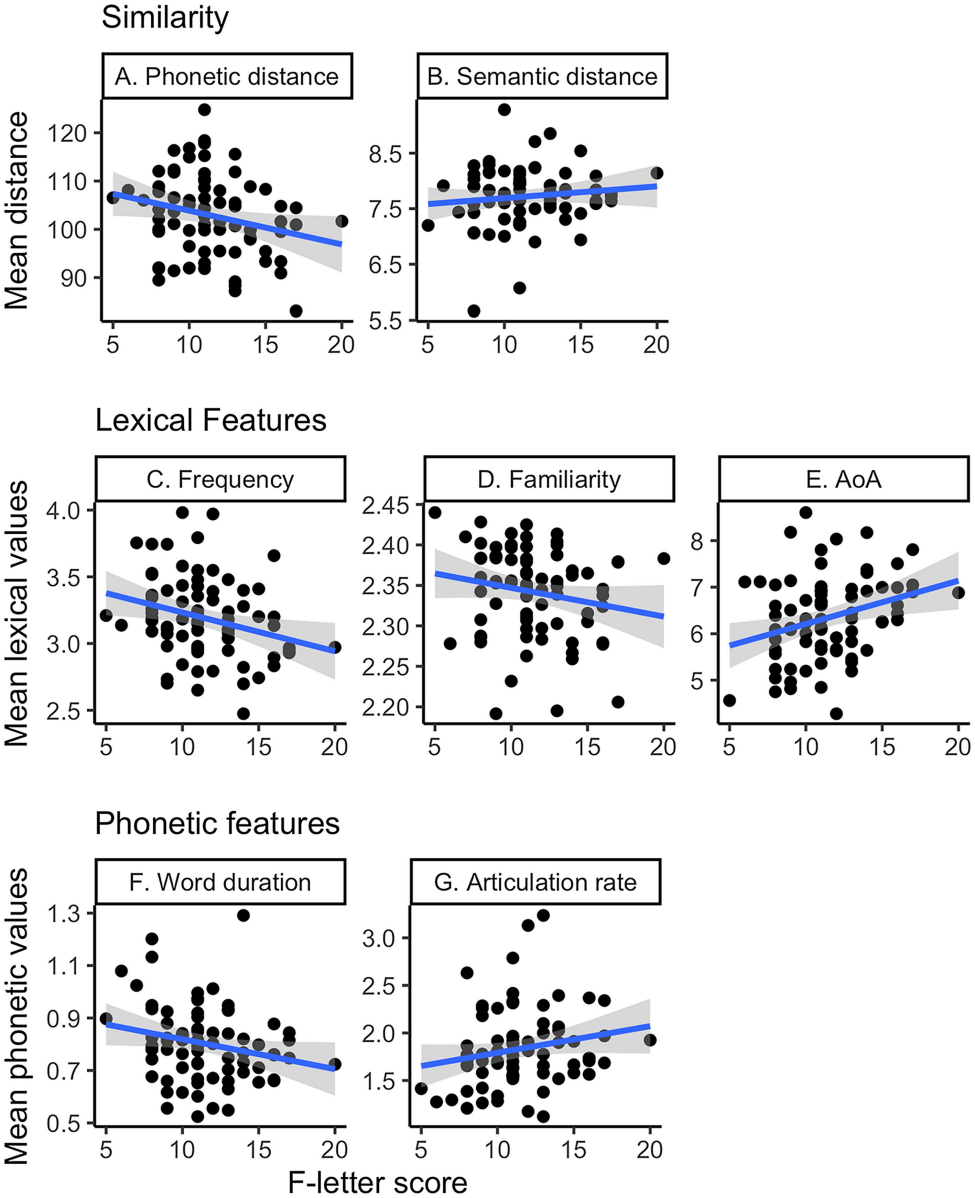
F-letter performance and the mean language measures. The x-axis shows the fluency score in all panels.

Higher F-letter scores were associated with smaller average phonetic distance (greater similarity) between words (*ρ* = −0.25, *p* = 0.033; Figure 2A), suggesting that speakers with a good performance produced words that were phonetically similar. However, semantic distance was not correlated with the F-letter scores (*ρ* = 0.05, *p* = 0.68; Figure 2B).

#### Lexical Features

F-letter score was negatively correlated with mean word frequency (*ρ* = −0.33, *p* = 0.003; Figure 2C) and familiarity (*ρ* = −0.24, *p* = 0.035; Figure 2D) and was positively associated with AoA (*ρ* = 0.38, *p* < 0.001; Figure 2E). Thus, speakers with a high F-letter score produced words that were less frequent, less familiar, and higher AoA than those who scored low. Neither concreteness nor semantic ambiguity was significantly correlated with the F-letter performance (Concreteness: *p* = 0.5; Ambiguity: *p* = 0.27; data not shown in Figure 2).

#### Phonetic Features

Higher F-letter scores were significantly associated with a faster articulation rate (*ρ* = 0.24, *p* = 0.034; Figure 2F) and a shorter spoken word duration (*ρ* = −0.26, *p* = 0.023; Figure 2G). However, we did not find any significant correlation between the number of phonemes/syllables and the F-letter scores (phonemes: *ρ* = 0.01, *p* = 0.9; syllables: *ρ* = 0.11, *p* = 0.33; data not shown in Figure 2).

#### Backward Selection Linear Model

**Figure 3:**
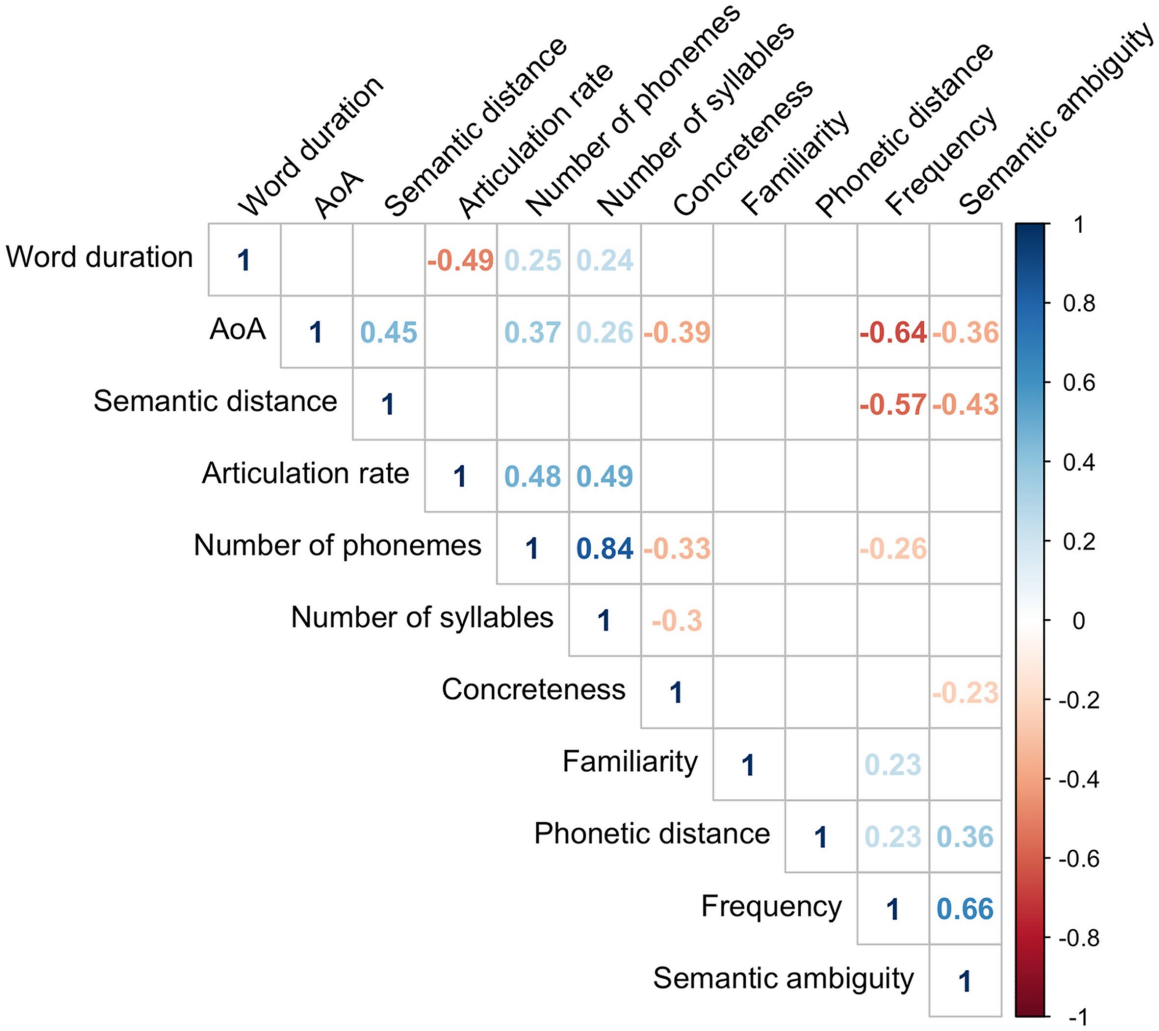
The correlation matrix of the mean values of all variables. Only significant correlations (*p* < 0.05) are shown in the figure.

To evaluate how the language variables were related with F-letter performance, we built a linear regression model where the dependent variable was F-letter score and the independent variables were the mean values of all language variables that showed significant correlations with F-letter performance (above). Table 2 summarizes the result of the best fit model (*R*^2^ = 0.26, *p* < 0.001); word duration, AoA, and phonetic distance survived elimination. The model estimated that 0.9 of the fluency score increased as the averaged AoA increased by 1 year, and fluency scores decreased by 0.09 when the averaged phonetic distance increases by one. Similarly, fluency scores decreased by 5.7 when word duration increased by 1 s. A correlation matrix among all explanatory variables is shown in Figure 3.

**Table 2:** Results of a linear regression model of the relation between F-letter scores and the average values of the selected language measures.

	Estimate	Std. Error	*t*-value	Pr(> |t|)
(Intercept)	19.425	5.129	3.787	0.000
AoA	0.908	0.340	2.669	0.009
Word duration	−5.729	2.124	−2.698	0.009
Phonetic distance	−0.089	0.037	−2.385	0.020

#### Individual Variance

**Figure 4:**
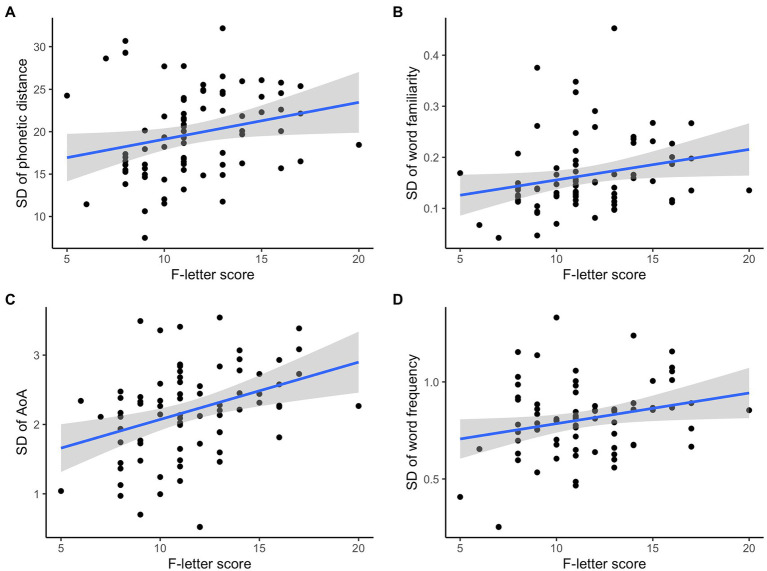
F-letter performance and *SD* of the language measures.

Higher F-letter scores were correlated with higher *SD* values in phonetic distance (*ρ* = 0.33, *p* = 0.004; Figure 4A), familiarity (*ρ* = 0.31, *p* = 0.007; Figure 4B), and AoA (*ρ* = 0.37, *p* < 0.001; Figure 4C). F-letter score was not significantly correlated with the word frequency (*ρ* = 0.22, *p* = 0.059; Figure 4D), or with other measures (all *p* > 0.1).

#### Backward Selection Linear Model of *SD* Values

**Figure 5:**
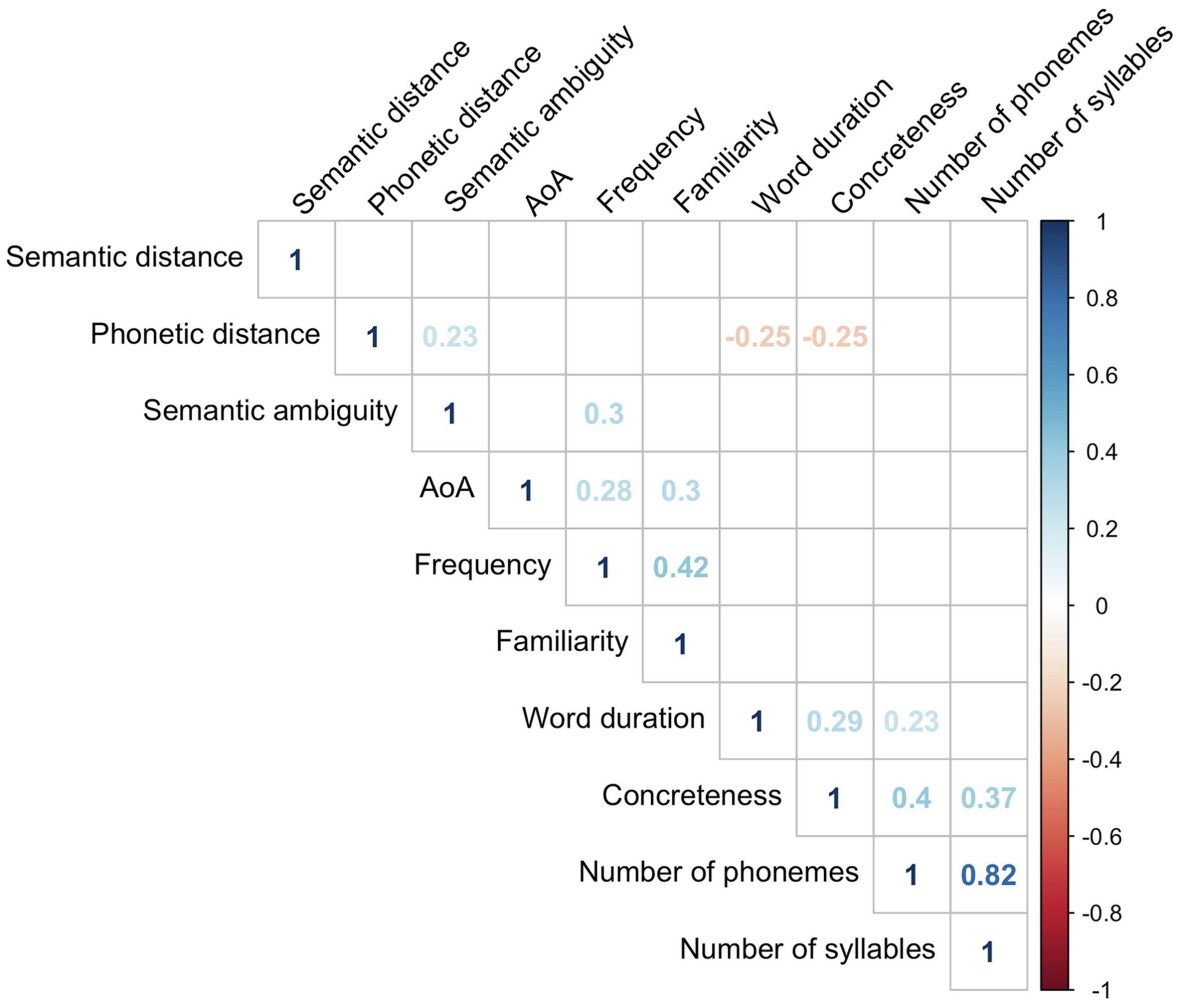
The correlation matrix of the *SD* values of all variables. Only significant correlations (*p* < 0.05) are shown in the figure.

Table 3 shows the results of the best fit linear regression model of the F-letter scores and *SD* values of the language measures. Final model fit was *R*^2^ = 0.21 (*p* < 0.001), with AoA and phonetic distance surviving elimination, suggesting that individuals with a higher variability in these measures had higher F-letter scores. A correlation matrix of the *SD* values of all variables is reported in Figure 5.

**Table 3:** Results of a linear regression model of the relation between F-letter score and *SD* of the selected language measures.

	Estimate	Std. Error	*t*-value	Pr(> |t|)
(Intercept)	4.560	1.635	2.789	0.007
AoA	1.724	0.462	3.729	0.000
Phonetic distance	0.158	0.059	2.667	0.009

### Response Time and the Language Measures

**Figure 6:**
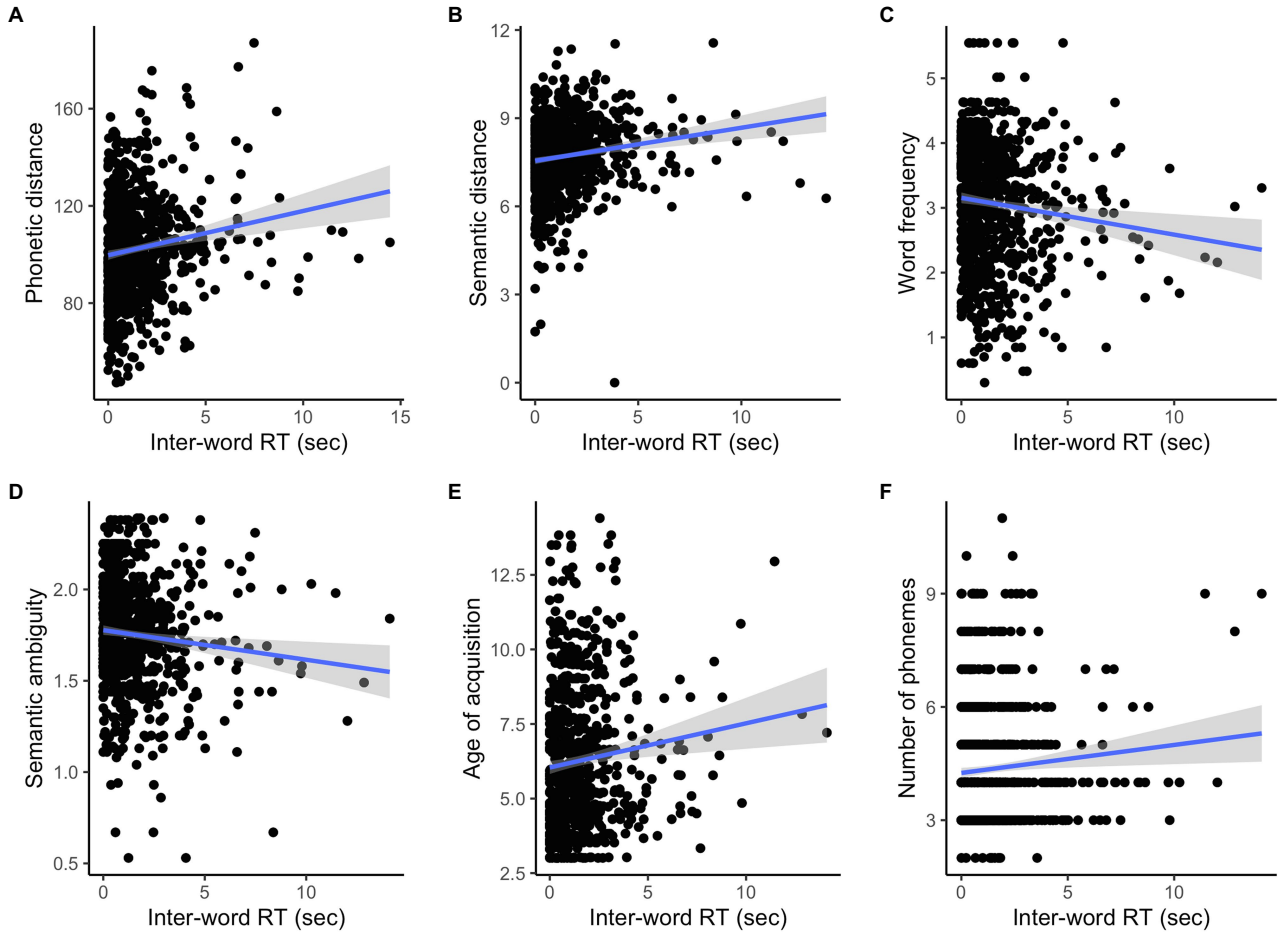
Inter-word RT and the language measures.

Table 4 shows the results of linear mixed-effects regression models for each language variable, and significant results are illustrated in Figure 6. Inter-word RT (RT after the initial word) increased when the following word was not phonetically or semantically similar to the previous word (*p* < 0.001 for both measures; Figures 6A,B). Duration of inter-word RT also increased when the following word was a less frequent (*p* = 0.002; Figure 6C), less ambiguous one (*p* = 0.006; Figure 6D), acquired later (*p* = 0.002; Figure 6E), or had more phonemes (*p* = 0.006; Figure 6F). Inter-word RT did not significantly vary by the other language measures (Table 4), and the first RT did not significantly vary by any language measures (*p* > 0.1 for all variables).

**Table 4:** Results of separate linear mixed-effects models of the relation between RT and the language measures.

	Estimate	Std. Error	*t*-value	Pr(> |t|)
Phonetic distance	0.013	0.003	4.282	0.000
Semantic distance	0.227	0.056	4.029	0.000
Word frequency	−0.211	0.067	−3.156	0.002
Semantic ambiguity	−0.651	0.232	−2.814	0.006
AoA	0.105	0.032	3.333	0.002
Number of phonemes	0.144	0.051	2.832	0.006
Number of syllables	0.179	0.096	1.865	0.067
Word duration	0.445	0.285	1.561	0.129
Concreteness	−0.068	0.068	−1.009	0.316
Familiarity	−0.268	0.332	−0.807	0.421

### Timing Within Task and the Language Measures

**Figure 7:**
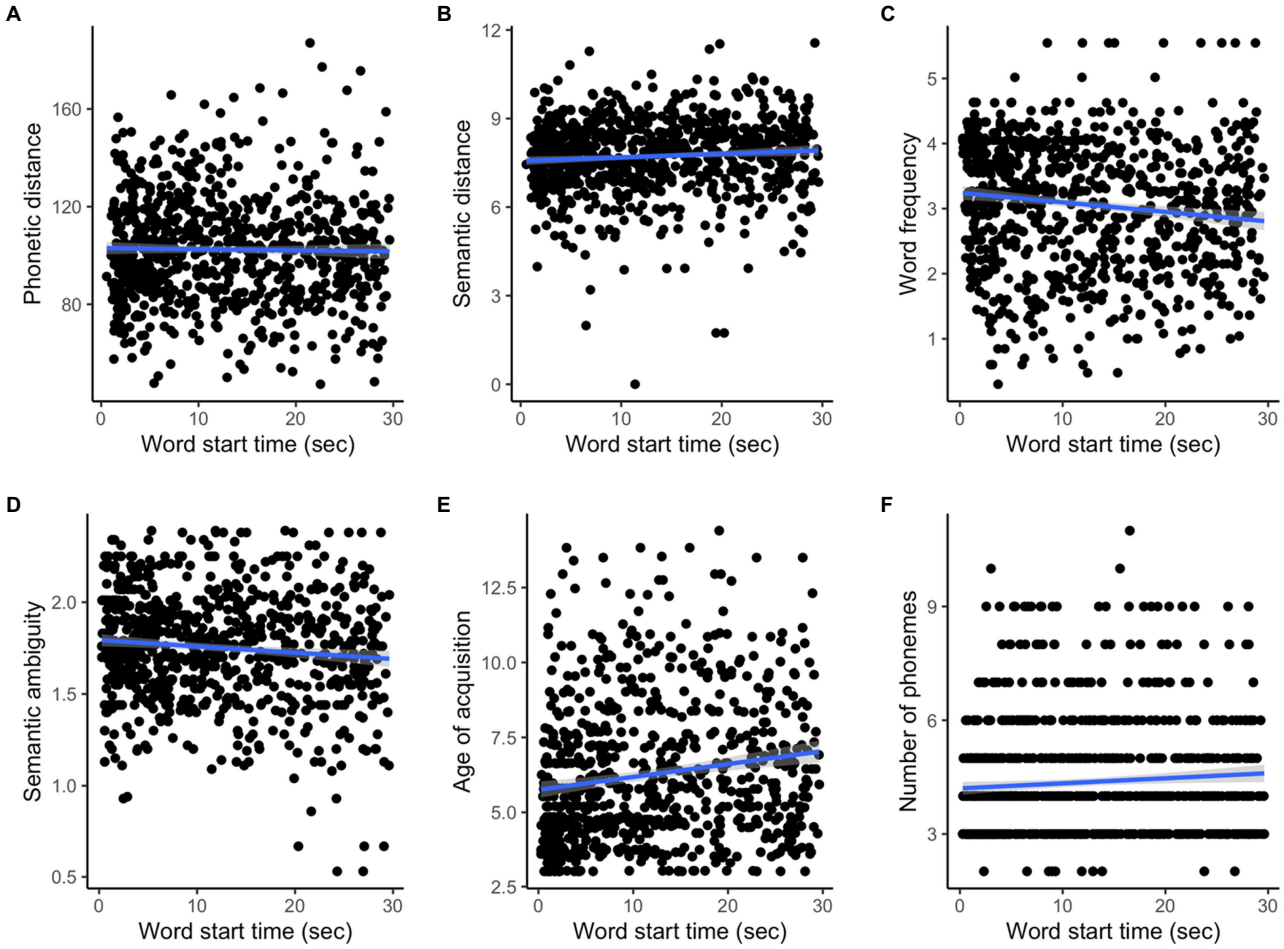
Changes of the language measures over task time.

Table 5 summarizes the results of multiple linear mixed-effects models that tested associations between timing within task and language measures. Phonetic distance did not change over time (Figure 7A), whereas semantic distance increased over time during the task (*p* = 0.031; Figure 7B). Word frequency and semantic ambiguity decreased over time during the task (frequency: *p* < 0.001; ambiguity: *p* = 0.003; Figures 7C,D), whereas AoA and number of phonemes increased (AoA: *p* < 0.001; number of phonemes: *p* = 0.045; Figures 7E,F). Other language measures did not change over time (Table 5).

**Table 5:** Results of separate linear mixed-effects models of the relation between task time and the language measures.

	Estimate	Std. Error	*t*-value	Pr(> |t|)
Word frequency	−1.227	0.307	−3.996	0.000
Semantic ambiguity	−3.066	1.031	−2.974	0.003
AoA	0.591	0.133	4.453	0.000
Number of phonemes	0.461	0.225	2.051	0.045
Number of syllables	0.518	0.446	1.162	0.250
Concreteness	−0.434	0.300	−1.446	0.153
Familiarity	−2.887	1.634	−1.767	0.084
Phonetic distance	−0.008	0.015	−0.513	0.610
Semantic distance	0.591	0.268	2.205	0.031

## Discussion

F-letter-guided fluency is a neuropsychological measure commonly used to assess individual working memory performance. In this study, we investigated how language characteristics of F-letter words were related with F-letter total score, RT, and timing within task using the F-letter fluency data produced by 76 young healthy participants. We manually corrected the automated POS and forced alignments to ensure and validate the accuracy of automated methods. Errors were minimal, and trained linguists corrected 1.2% of POS tagging and 7.2% of forced alignments. These findings suggested that automated methods could be used in the place of more labor-intensive manual methods. Our data showed in that (1) the first and mean inter-word RTs were negatively correlated with the F-letter total score, (2) the number of F-letter words produced decreased over time, and (3) inter-word RT increased over timing within task. We showed that the mean word frequency, familiarity, AoA, word duration, phonetic similarity, and articulation rate were significantly correlated with F-letter scores. When all significant variables were considered together, only the mean values of AoA, word duration, and phonetic similarity were significantly related with the F-letter performance. The *SD* values of AoA, familiarity, and phonetic similarity were also significantly correlated with the F-letter scores, suggesting that speakers who had high variance in those measures scored high in the letter fluency task. When all *SD* values of the significant measures were considered, the *SD* values of AoA and phonetic distance were significantly related with the F-letter scores. Also, word frequency and semantic ambiguity were negatively correlated with inter-word RT, whereas AoA, number of phonemes, phonetic distance, and semantic distance were positively correlated with inter-word RT. None of our language variables was related with the first RT. Furthermore, word frequency, semantic ambiguity, AoA, number of phonemes, and semantic distance significantly changed over time during the task. Some of these characteristics have been described in the previous studies (Rohrer et al., 1995; Crowe, 1998; Fernaeus and Almkvist, 1998; Luo et al., 2010; Sauzéon et al., 2011; Shao et al., 2014). However, no previous study has been able to capture the broad scope of performance that we describe here, nor examine the relationships between all of these measures. This was possible because of our automated analysis of digitized speech. In this study, we emphasize the usefulness of this analytic approach in the assessment of F-letter performance, and potentially other verbal neuropsychological measures. In the F-letter fluency task, this is reflected by the consistently significant role played by the phonetic properties of words.

Our automated results agree with previous manual assessments of fluency tasks. Raskin et al. (1992) and Troyer et al. (1997) showed that the switching ability between subcategory clusters in a fluency task is highly correlated with fluency performance. For example, in a letter fluency data, speakers who can swiftly switch from one group of phonetically similar words to another word that is phonetically distinct from the previous group in the articulatory space tended to show high performance. Similarly, in our results, the *SD* values of phonetic distance were significantly correlated with the F-letter scores, which suggests that speakers who produced words that highly varied in the articulatory space scored high in the task. This makes an interesting comparison with the average and *SD* values of semantic distance in our result, which were not correlated with the F-letter scores. It is in line with the previous finding that only switches between phonemic subcategories are important in a letter fluency data (Troyer et al., 1997). This may be due in part to the possibility that switching is an executive function more accurately reflected in vocabulary tokens that do not have a semantic link.

In addition, the phonetic and semantic distances showed interesting results with respect to RT and task timing. Inter-word RT increased when the phonetic or semantic distance between two F-letter words increased, which suggests that a longer processing time was required to retrieve a word from the mental lexicon that was not phonetically or semantically similar to the previous word (e.g., switching subcategories). Interestingly, the semantic distance increased over time during the task, whereas the phonetic distance did not change throughout the task. Previous studies (e.g., Luo et al., 2010; Katzev et al., 2013; Shao et al., 2014) note that a letter fluency task requires a novel word retrieval strategy that is rarely used in daily life. In everyday speech production, participants generate meaningful messages to communicate with, influence, demonstrate identity with and posture toward each other, which requires retrieving words that are semantically relevant. However, since participants are asked to list words that are phonetically related in the F-letter fluency test, they need to suppress the activation of semantically related words to successfully complete the test. This may explain why we observed that semantic distance increased over time, whereas the phonetic distance remained unchanged, suggesting that participants were able to successfully suppress the activation of semantic relations between words. We plan to analyze semantically guided category fluency data in the near future to investigate whether the same or different trends are observed when emphasizing a semantic target. Future research is needed to examine if patients with neurodegenerative disease or psychosis can successfully suppress semantically related words in a letter fluency data, compared to healthy controls.

We found that higher mean and *SD* values of AoA were significantly related with higher F-letter scores in our results. These findings suggest that a broader vocabulary, reflected by a later average AoA and broader AoA *SD*, supports better performance on the F-letter fluency task. The relation of AoA and fluency performance will need to be further studied with wide age and education ranges in both letter and category tasks. Nevertheless, this has important implications for clinical populations. Juhasz et al. (2012) showed that word produced by schizophrenia patients had lower AoA values on average than those generated by healthy controls in a semantically guided category fluency task, but not in a letter fluency task, even though patients scored significantly lower than controls in both fluency tasks. Forbes-Mckay et al. (2005) observed that patients with AD produced words that were acquired earlier (lower AoA) than healthy controls in a semantically guided category fluency task, consistent with AD patients’ loss of knowledge for words acquired later in life. These findings are consistent with the idea that performance on the F-letter-guided fluency task is relatively sensitive to the breadth of vocabulary.

Inter-word RTs were related with total score and several language variables, showing that it took longer to produce words that were less frequent, less ambiguous, older AoA, and more phonemes. In contrast, the first RT was not related with any language measure but was related to total score. Participants are generally not given adequate instructions to test the first RT, including a warning signal for initiating performance, and an indication of the criterion for performance (e.g., words beginning with the letter “f”). In our study, the start of the task was initiated by the participants, and the participants were not aware of what letter they were given before the task. We used these parameters in our task since this is the most common way in which this task is administered. However, the relation of language characteristics, first RT, and fluency performance needs to be further validated with future studies that control task initiation.

In our results, participants with high scores tended to speak more quickly, evidenced by faster articulation rates and shorter word durations. Importantly, articulation rate was calculated without pause duration, and the number of phonemes was not correlated with F-letter performance, which suggests that the shorter word duration of high performers cannot be easily explained by their producing words shorter in length. High performers also had shorter inter-word RTs, indicating that these subjects retrieved words quickly from their mental lexicon. The significant correlations of word duration and articulation rate with F-letter performance have methodological implications to consider for measuring latency or RT in fluency data. Inter-word latency or RT in the literature has been previously defined as the duration from the onset of the first word to the onset of subsequent words; however, word duration itself may confound RT measures. In particular, when comparing patients to healthy controls, differences in word duration and articulation rate need to be taken account, since patients with neurodegenerative disease usually speak slowly and score low in fluency tasks compared to healthy controls (e.g., Ash et al., 2009; Boschi et al., 2017).

The results we described in this paper were possible only because of our capacity for automated analysis of digitized speech. Our previous automated analyses of digitized speech samples of the Cookie Theft picture description task have provided novel acoustic and lexical properties in our published work of brief picture descriptions (Nevler et al., 2017, 2019; Cho et al., 2021a,b), including automated diagnostic categorization at >90% accuracy of patients with neurodegenerative disease (Cho et al., 2020). This study enhances the informativeness of our automated analysis pipelines by showing that it can be applied to a traditional neuropsychological task and support patients with neurodegenerative disease by optimizing diagnostic value with less patient burden through briefer assessments, monitoring disease progression more reliably, and providing prognostic information with improved validity. Automated methods capture subtle differences to support these outcomes, and moreover, while manual assessments of performance are variable, even among experts, fully automated analyses will further improve objectivity and reliability.

## Conclusion

The present study showed that fully automated analyses of letter fluency data yield detailed metrics of word production and show that F-letter performance is associated with acoustic, semantic, and lexical features of the words produced. We demonstrated that (1) automated methods we used in this study are compatible with manual methods, (2) they are faster, thus more scalable, and (3) because they are objective, they offer the promise of improving comparability and reproducibility by removing variation in practice across studies. We believe the method proposed in this work captures subtle and rich language characteristics during test performance that cannot be extracted manually without massive effort. This method can be easily applied for letter-guided category fluency production similarly acquired in neurodegenerative patients. In the near future, we plan to analyze category fluency data produced by healthy controls to compare the results with the present letter fluency data and plan to analyze healthy speakers in various age and education ranges to further investigate the effect of age and education in the language measures and build a diverse normative database of both letter- and category-guided fluency tasks that can also be used in the study of patients.

## Data Availability Statement

The raw data supporting the conclusions of this article will be made available by the authors, without undue reservation.

## Ethics Statement

The studies involving human participants were reviewed and approved by the Institutional Review Board at the University of Pennsylvania. The patients/participants provided their written informed consent to participate in this study.

## Author Contributions

SC, MG, ML, and KC developed the research concept and design. ML and CC collected the data as part of a larger project. SC analyzed the data and drafted the manuscript. All authors worked together on the interpretation of the results, framing the manuscript, and revising it.

## Conflict of Interest

The authors declare that the research was conducted in the absence of any commercial or financial relationships that could be construed as a potential conflict of interest.

## Publisher’s Note

All claims expressed in this article are solely those of the authors and do not necessarily represent those of their affiliated organizations, or those of the publisher, the editors and the reviewers. Any product that may be evaluated in this article, or claim that may be made by its manufacturer, is not guaranteed or endorsed by the publisher.

## Footnotes

^1^ https://nlp.stanford.edu/projects/glove/
