# Supplementary material for: Automated Analysis of Digitized Letter Fluency Data
Source: Front Psychol. 2021 Jul 29;12:654214. doi: 10.3389/fpsyg.2021.654214 (PMC8359864; doi:10.3389/fpsyg.2021.654214)
Supplement: Supplementary file 1 [file Table_1.DOCX]

Supplementary Material

**
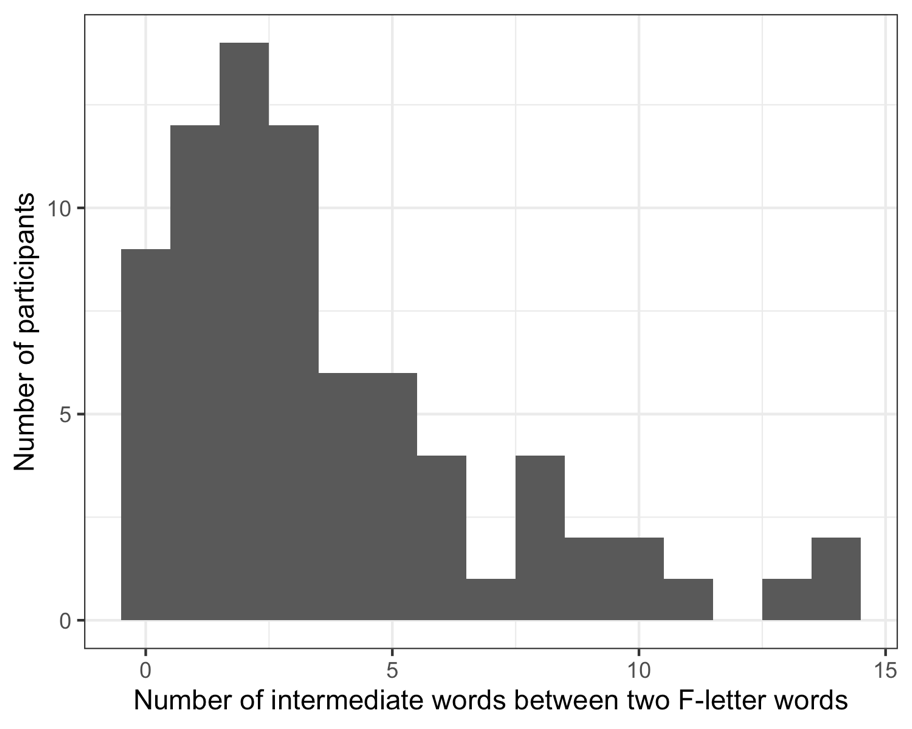
**

**Supplementary Figure 1.** Number of participants by the number of intermediate words between two consecutive F-letter words.
